# Molecular Characterization of Two Lysophospholipid:acyl-CoA Acyltransferases Belonging to the MBOAT Family in *Nicotiana benthamiana*

**DOI:** 10.1371/journal.pone.0144653

**Published:** 2015-12-18

**Authors:** Donghui Zhang, Katarzyna Jasieniecka-Gazarkiewicz, Xia Wan, Ling Luo, Yinbo Zhang, Antoni Banas, Mulan Jiang, Yangmin Gong

**Affiliations:** 1 Key Laboratory of Biology and Genetic Improvement of Oil Crops, Ministry of Agriculture, Oil Crops Research Institute of Chinese Academy of Agricultural Sciences, Wuhan, 430062, China; 2 Intercollegiate Faculty of Biotechnology of University of Gdansk and Medical University of Gdansk, 80–822, Gdansk, Poland; University Paris South, FRANCE

## Abstract

In the remodeling pathway for the synthesis of phosphatidylcholine (PC), acyl-CoA-dependent lysophosphatidylcholine (lysoPC) acyltransferase (LPCAT) catalyzes the reacylation of lysoPC. A number of genes encoding LPCATs have been cloned and characterized from several plants in recent years. Using Arabidopsis and other plant LPCAT sequences to screen the genome database of *Nicotiana benthamiana*, we identified two cDNAs encoding the putative tobacco LPCATs (NbLPCAT1 and NbLPCAT2). Both of them were predicted to encode a protein of 463 amino acids with high similarity to LPCATs from other plants. Protein sequence features such as the presence of at least eight putative transmembrane regions, four highly conserved signature motifs and several invariant residues indicate that NbLPCATs belong to the membrane bound *O*-acyltransferase family. Lysophospholipid acyltransferase activity of NbLPCATs was confirmed by testing lyso-platelet-activating factor (lysoPAF) sensitivity through heterologous expression of each full-length cDNA in a yeast mutant Y02431 (*lca1*△) disrupted in endogenous LPCAT enzyme activity. Analysis of fatty acid profiles of phospholipids from the NbLPCAT-expressing yeast mutant Y02431 cultures supplemented with polyunsaturated fatty acids suggested more incorporation of linoleic acid (18:2n6, LA) and α-linolenic acid (18:3n3, ALA) into PC compared to yeast mutant harbouring empty vector. *In vitro* enzymatic assay demonstrated that NbLPCAT1had high lysoPC acyltransferase activity with a clear preference for α-linolenoyl-CoA (18:3), while NbLPCAT2 showed a high lysophosphatidic acid (lysoPA) acyltransferase activity towards α-linolenoyl-CoA and a weak lysoPC acyltransferase activity. Tissue-specific expression analysis showed a ubiquitous expression of *NbLPCAT1* and *NbLPCAT2* in roots, stems, leaves, flowers and seeds, and a strong expression in developing flowers. This is the first report on the cloning and characterization of lysophospholipid acyltransferases from *N*. *benthamiana*.

## Introduction

Phospholipids play important roles in almost all organisms. They are not only structural and functional components of biological membranes but also precursors of many lipid mediators such as platelet-activating factor (PAF) and eicosanoids[1]. There are different species of phospholipids that can be distinguished from each other in term of the phosphoryl head groups and the fatty acids with different chain lengths and degrees of saturation [2]. Cell membranes from different organisms contain several classes of phospholipids, such as phosphatidylcholine (PC), phosphatidylethanolamine (PE), phosphatidic acid (PA), phosphatidylserine (PS), phosphatidylinositol (PI), and phosphatidylglycerol (PG). Starting with phosphatidic acid, phospholipids are formed via the *de novo* pathway [3], and subsequently undergo remodeling pathway [4] that is mediated by a phospholipid lipase A_2_ and a lysophospholipid acyltransferase (LPLAT). To date, very limited numbers of LPLAT proteins have been identified and characterized although the phospholipid remodeling pathway takes place in almost all organisms. The nomenclature of LPLAT has been proposed based on their substrate specificities [5]. For example, LPCAT enzyme catalyzes the formation of PC using lysoPC as preferred substrate, while LPAAT exhibits high acyltransferase activity towards lysoPA.

Among various species of phospholipids, PC is one of the major and essential structural components of cell membrane. In plant cells, besides the fundamental role in the formation of biomembrane, PC does not only serves as a fatty acyl donor for neutral lipid biosynthesis, but also as a major acyl carrier used by desaturases and hydroxylases for fatty acid desaturation and hydroxylation [6,7].In most eukaryotes, a *de novo* pathway called the Kennedy pathway [3] exists for synthesizing PC, in which PC is formed directly from diacylglycerol (DAG) and CDP-choline in a reaction catalyzed by diacylglycerol:cholinephosphotransferase (CPT; EC 2.7.8.2). However, the final acyl groups of PC are distributed in an asymmetric manner within the cell. Previous studies in plants and animals suggest that the *sn*-1 position of PC is frequently occupied by saturated and monounsaturated fatty acids, whereas polyunsaturated fatty acids are usually esterified at the *sn*-2 position [5,8–10]. It has been known that the appropriate fatty acyl composition of PC is acquired by further remodeling, and it was estimated that more than 50% of PC is remodeled after its *de novo* synthesis [11]. PC remodeling takes place through deacylation and reacylation processes that is defined as the Lands cycle [12]. In the remodeling pathway, PC is first deacylated at the *sn*-2 position to generate lysophosphatidylcholine (lysoPC) by the action of phospholipase A2, followed by the reacylation of lysoPC to synthesize PC that is catalyzed by acyl-CoA-dependent lysophosphatidylcholine acyltransferase (LPCAT; EC2.3.1.23). Rapid and extensive turnover of the *sn*-2-acyl moiety of PC has been reported in yeast [13], plants [14] and animals [15].PC remodeling is important for the integrity of cell membranes, lipid biosynthesis, vesicle trafficking, and other related biological functions.

Despite the discovery of the Lands cycle 50 years ago, LPCAT enzymes that mediate the reacylation of lysoPC in the remodeling pathway were identified in recent years. Lca1p, which exhibits a lysoPC substrate preference and displays particularly high activities for C_16_ and C_18_ saturated and monounsaturated fatty acyl-CoAs, was reported to represent a major LPCAT enzyme involved in PC turnover process in yeast [13].The cDNAs encoding LPCAT enzymes from several plant species, including *Arabidopsis thaliana* [16], *Brassica napus* [17], *Ricinus communis* [18], were also cloned and characterized. It was found that these plant LPCATs prefer lysoPC as a substrate over other lysophospholipid classes and exhibit a preference for C_16_ and C_18_-unsaturated acyl-CoAs, and they are thought to participate in the Lands cycle. Up to now, four LPCATs (LPCAT1-4) have been identified in both mouse and human. In animals, both LPCAT1 and LPCAT2 catalyze not only PC synthesis (LPCAT activity) but also PAF (platelet-activating factor) synthesis (lyso-PAF acetyltransferase). They have distinct expression patterns, and participate in two kinds of PAF remodeling pathways in inflammatory cells: LPCAT2-mediated inflammatory/inducible pathway and LPCAT1-mediated non-inflammatory/constitutive remodeling pathway [19–22]. LPCAT3 and LPCAT4 show different activities for fatty acyl-CoAs, with the former preferring polyunsaturated fatty acyl-CoAs and the latter preferring 18:1-CoA. Both of them catalyze the synthesis of not only PC but also other phospholipids (such as PE and PS) [11,23]. The existence of multiple LPCAT acyltransferases in animal cells may act to incorporate different fatty acyl moieties into phospholipids and contribute to the diversity of membrane composition.

In plant oilseeds that were engineered to produce omega-3 polyunsaturated fatty acids (such as eicosapentaenoic acid (EPA, 20:5) and docosahexaenoicacid (DHA, 22:6)) by introducing additional desaturases and elongases, LPCAT-catalyzed reaction was considered to be a possible metabolic bottleneck to accumulate high-levels of target fatty acids [7,24]. This bottleneck is probably caused by substrate dichotomy: in omega-3 polyunsaturated fatty acid (PUFA) biosynthetic pathway, fatty acid desaturases typically use PC as a substrate whereas elongases use acyl-CoAs. Thus, synthesis of polyunsaturated fatty acids requires efficient shuttling of fatty acid intermediates between PC and acyl-CoA pools. Addressing whether LPCAT mediates fatty acid flux in transgenic plants is, however, hampered by the lack of detailed analysis of LPCATs cloned from plants. In such a project we use *Nicotiana benthamiana*as heterologous expression host to determine the proposed role of LPCATs in mediating fatty acid flux. Assembly of multistep omega-3 PUFA biosynthetic pathway has been achieved in both of these two expression hosts [25,26]. The clear genetic background, availability of multiple molecular toolboxes, establishment of leaf-based transient expression system and seed-specific expression system allow *N*. *benthamiana* to serve as a model plant to manipulate complex metabolic pathways and explore whether LPCAT-catalyzed reaction is a metabolic bottleneck in transgenic synthesis of omega-3 PUFAs in plants. Here we report the identification of two *N*. *benthamiana* LPLATs, both of which possess LPCAT activity using 18:3-CoA as the best donor. This study provides molecular basis for solving the metabolic bottleneck possibly caused by LPCAT activity in transgenic plants as well as for determining the role of LPCATs in lipid synthesis in tobacco.

## Materials and Methods

### Plant, microorganism materials and growth conditions

*Nicotiana benthamiana* was grown under 180 μM photons/m^2^/s (16 h light/8 h dark) at 22°C. Plants were used for extraction of total RNAs when they were 10 to 14 d old. Plant materials for gene expression profiling were obtained by germinating *N*. *benthamiana* seeds in petri dishes containing one-half Murashige and Skoog medium with 3% sucrose for 14 d. The seedlings were then transferred into glass flasks with solid medium containing Murashige and Skoog medium and 3% sucrose and grown at 22°C. Plant tissues grown at different developmental stages were harvested, immediately frozen in liquid N_2_ and stored at -80°C. Total RNA was extracted using Trizol reagent (Invitrogen) from these samples and used for quantitative reverse-transcription (RT) PCR.

Yeast strain Y02431 (*lca1*△, *MATa*, *his3*△1, *leu2*△0, *met15*△0, *ura3*△0, *YOR175c*::*KanMX4*), kindly provided by Dr. JitaoZou (Plant Biotechnology Institute, National Research Council Canada, Saskatoon), was used to test LPCAT activity by heterologous expression. Yeast cells were maintained on YPD plates (1% yeast extract [w/v], 2% peptone [w/v], and 2% glucose [w/v]) solidified with 2% agar (w/v). For heterologous expression of NbLPCAT1 and NbLPCAT2, the overnight cultures that were inoculated by single colonies were transferred into synthetic complete-ura (SC-Ura) medium containing 2% glucose and grown for 3 d at 28°C and 180 rpm in an orbital shaker. Yeast cells were harvested by centrifugation, washed with sterile distilled water, and transferred to SC-Ura medium containing 2% galactoseand 1% raffinose to induce the expression of NbLPCAT genes for additional 24 h at 28°C. To determine substrate preference of NbLPCATs for polyunsaturated fatty acids, supplementation of SC-Ura cultures with linoleic acid (18:2n6, LA) and α-linolenic acid (18:3n3, ALA) was carried out with 500 μM of the appropriate fatty acid in the presence of 1% (w/v) Tween 40 (Sigma-Aldrich). Cells were harvested by centrifugation (1,500×*g* for 5 min), washed three times (0.2% Tween 40, 0.1% Tween 40 and distilled water), freeze-dried, and used for lipid analysis.

### Gene cloning and sequence analysis

Database (http://sydney.edu.au/science/molecular_bioscience/sites/benthamiana/) search was performed using the Basic Local Alignment Search Tool (BLAST) program with sequences derived from conserved regions of the Arabidopsis LPCAT acyltransferases and other acyltransferases of the membrane bound *O*-acyltransferase (MBOAT) family. Two genes that encode polypeptides composed of 463 amino acids were identified as the hypothetical *N*. *benthamiana* LPCAT-coding sequences. These sequences, designated *NbLPCAT1* and *NbLPCAT2*, were amplified using single-strand cDNAs transcribed from total RNA of tobacco leaves. The primers used for PCR were derived from *N*. *benthamiana* genomic sequences and listed in (Table 1). The generated double-stranded cDNAs were cloned into pEASY^™^-T1 Simple vector, sequenced, and compared with the corresponding genomic sequences.

**Table 1:** Primers used in this study.

Primers	Sequences
NbLPCAT 1F	5' GCGGCCGCACATAATGGAGCTGCCGGAGATGGAAT 3'
NbLPCAT 1R	5' TTAATTAATCACTCTTCTTTCTTAGCTTTAGATC 3'
NbLPCAT 2F	5' GCGGCCGCACATAATGGGGCTGCCGGAGATG 3'
NbLPCAT 2R	5' TTAATTAATCACTCTACTTTCCTAACTTTAG 3'
RT- NbLPCAT1F	5' TTTGCTCAAGTTGCCATCTG 3'
RT- NbLPCAT1R	5' TCTCTCCGTCCAGTCAAGGT 3'
RT-Nblpcat2F	5' GTTCTCTCTCGCCGTTTCTG 3'
RT-Nblpcat2R	5' TCCCGTAGCATCAATTCCTC 3'
EF-1α-F	5' TGAGATGCACCACGAAGCTC 3'
EF-1α-R	5' CCAACATTGTCACCAGGAAGTG 3'

Sequences homologous to the predicted sequences for *N*. *benthamiana* LPCATs were retrieved using the BLASTP program (www.ncbi.nlm.nih.gov). The amino acid sequences for LPCAT proteins deposited at GENBANK were aligned using the Clustal X V.1.83 program under the default settings. The phylogenetic tree was constructed with the neighbor-joining method and evaluated with 1,000 rounds of bootstrapping using the MEGA 5.0 program [27].

### Construction of plasmids

The *N*. *benthamiana* LPCAT1 and LPCAT2 ORFs were amplified from total RNA of plant leaves using primer pairs NbLPCAT1F/R and NbLPCAT2F/R that introduced in-frame *Not*I or *Pac*I site 5' and 3' of the start and stop codons of NbLPCAT1 and NbLPCAT2, respectively. The amplicons were gel-purified, cloned into pEAST^™^-T1 simple vector and sequenced. NbLPCAT1 and NbLPCAT2 ORFs were released from the corresponding T-vector by digestion of *Not*I and *Pac*I, and ligated into the yeast expression vector pESC-Ura, which was also digested by *Not*I and *Pac*I. The resulting plasmids were referred to as pESC-Ura/NbLPCAT1, pESC-Ura/NbLPCAT2 and empty vector pESC-Ura, respectively. Yeast expression plasmids containing NbLPCAT1 or NbLPCAT2 ORFs or empty vector (pESC-Ura) were transformed into the yeast mutant Y02431 by the standard lithium acetate procedure [28]. Transformants were selected by growth on synthetic complete medium (2% glucose [w/v], 0.67% yeast nitrogen base without amino acids) containing appropriate auxotrophic supplements but lacking uracil (SC-Ura). The single colonies were transferred into liquid SC-ura with 2% glucose and grown at 28°C overnight.

### Testing Lyso-PC sensitivity in the yeast *lca1* △mutant

Each transformant harboring the empty vector pESC-Ura or the plasmid for the expression of NbLPCAT was suspended in sterile distilled water and adjusted to an OD_600_ of 2, 1, 0.5, and 0.1. The resulting 2 μL yeast solution was spotted on an SC-Ura agar plate lacking uracil but containing 2% galactose and 1% raffinose and (a) 5 μg/mL lysoPAF; (b) 10 μg/mL lysoPAF; (c) 25 μg/mL lysoPAF; and (d) 30 μg/mL lysoPAF. Cell growth was evaluated after 72 h at 28°C to examine the lysoPC sensitivity for the growth of yeast *lca1*△mutant transformants.

*In vitro* acyltransferase activity assays Yeast cells (transformed either with empty plasmid or one of the tested LPCAT genes) were cultured for 24 h on an orbital shaker (220 rpm) at 30°C in synthetic uracil drop-out medium containing 2% glucose. After that time galactose was added to the final concentration of 2% (w/v) and cells were grown for additional 24 h. Microsomal fractions were prepared according to the method described by Dahlqvist et al. [29]. The yeast cultures (100 mL) were centrifuged and washed twice with distilled water. Each pellet was suspended in the 1 mL of ice-cold buffer (20 mMTris/HCl, pH7.9, 10 mM MgCl_2_, 1 mM EDTA, 5% (v/v) glycerol, 0.3 M ammonium sulphate) containing protease inhibitors (Complete, Roche Applied Sciences) and transferred to 2 mL Eppendorf tube with 1 mL glass beads (0.45–0.5 mm in diameter). The tubes were shaken (10 times, 30s) using the Mini Bead Beater-8 (Biospec Products, Bartlesville, OK, USA) and the homogenates were centrifuged for 10 min at 1,500 ×*g*. The resulting supernatants were transferred to the new tubes, and centrifuged at 100,000 × *g* for 2 h. The pellets were suspended in the 0.1 M potassium phosphate buffer (pH7.2) and these extracts, subsequently referred to as microsomal fractions or microsomes, were stored at -80°C until use for further analyses. All steps were performed at 4°C during the preparation of microsomal fractions.

In the assays measuring activity and determining substrate specificity (towards different fatty acyl acceptors and fatty acyl donors) of the tested lysophospholipid:acyl-CoA acyltransferases, the reaction mix contained 5nmol of [^14^C]-labelled fatty acyl-CoA, 5 nmol *sn*-1-18:1-lysophospholipid (LysoPC, LysoPA, LysoPE, or LysoPS), and 8 μg of microsomal proteins in 100 μL of 40 mM potassium buffer (pH 7.2). The reaction was carried out in Thermomixer Compact (Eppendorf) at 30°C with shaking (1250 rpm) for different times, and was stopped by addition to the assays of 375 μL chloroform/methanol (1:2; v/v), 5 μl acetic acid, 125 μL chloroform, and 125 μL water. After vigorous shaking, the test tubes were centrifuged (1000 × *g*) for 2 min. The phospholipids were extracted tothe chloroform fractions and separated by TLC on silica gel 60 plates (Merck, New York, USA) using chloroform/methanol/acetic acid/water (85/15/10/3.5, v/v/v/v) as the solvent system. Radioactive phospholipids were visualised and quantified on the plate by electronic autoradiography (Instant Imager, Packard Instrument Co.).

### Analysis of tissue-specific LPCAT gene expression

The tissue-specific expression patterns for NbLPCAT1 and NbLPCAT2 were analyzed by real-time quantitative PCR. Total RNA was extracted from developing seeds, 2-d-old germinating seedlings, roots, young leaves, stems and flowers. RNA samples were treated with DNase I (Takara) to remove contaminating DNA. First-stand cDNA was synthesized with the PrimeScript RT reagent Kit (Takara), using equal amounts of oligo (dT) and random primers, according to the manufacturer’s instructions. All Real-time reactions were performed in a MiniOpticon Thermal Cycler (Bio-Rad) detection system using the intercalation dye SYBR Green I master mix kit (Takara) as a fluorescent reporter.

PCR reaction was performed in triplicate in 20-μL volumes using 0.6 μL of each forward and reverse primer (10 μM), 10 μL SYBR Green master mix, 2 μL of cDNA and 6.8 μL of DEPC-treated water. Reactions were performed in 48-well plates (Bio-Rad) covered with optical adhesive covers (Bio-Rad). The amplification conditions were as follows: 95°C 15 min, and 40 cycles of 95°C for 10 s, 60°C for 20 s, and 72°C for 30 s; 72°C for 5 min.

The quantification of PCR products was performed via a calibration curve procedure using the transcript of Elongation factor 1α as an internal standard. PCR products were analyzed using melting curves (62°C to 95°C with increment of 0.5°C for 5 s) as well as agarose gel electrophoresis to ensure single product amplification. The ratio of gene-specific expression to Elongation factor 1α signal was defined as relative expression. Primers for specific amplification of each cDNA were designed using the online primer design software.

### Lipid extraction, separation and fatty acid analysis

Phospholipids were extracted from lyophilized yeast cells (about 0.1 g) using the method described by Gu et al. [30], and dissolved in 150μL chloroform. To separate phospholipids into different lipid classes, one-dimension thin-layer chromatography (TLC) was performed as described [31]. Briefly, phospholipid samples were spotted on silica gel plates (Merck) and then separated using a mobile phase of chloroform: ethanol: water: triethylamine (30:35:7:35, v/v/v/v). Lipid spots were visualized under UV light by staining with 0.1% (w/v) 2,7-dichlorofluorescein (Sigma) in 95% methanol, and identified by comparison with retention factor values of lipid standards (Sigma). After separation individual lipids were extracted from the Silica gel by using 3mL chloroform: methanol (1:1, v/v). Lipids were then dried under N_2_ and dissolved in 100 μL chloroform.

Fatty acids in each phospholipid class were methylated using 3 mL of 5% H_2_SO_4_ in methanol for 1 h at 90°C (including standard amounts of 22:0). The reaction was stopped by the addition of 2mL 0.9% NaCl and 3mL hexane. The hexane phase was analyzed by gas chromatography equipped with a flame ionization detector (FID) and an HP-FFAP capillary column (Agilent Technologies, 30m×250μM x 0.25μm). Methylated fatty acids were identified by chromatographic comparison with authentic standards (Sigma) and quantified by the surface peak method using 22:0 fatty acid as internal standard. Extraction and quantification were carried out in triplicate. Percentage of each FAME was calculated based on its amount in individual lipid class.

## Results and Discussion

### The tobacco LPLATs, conserved motifs and evolutionary relationships

**Fig 1:**
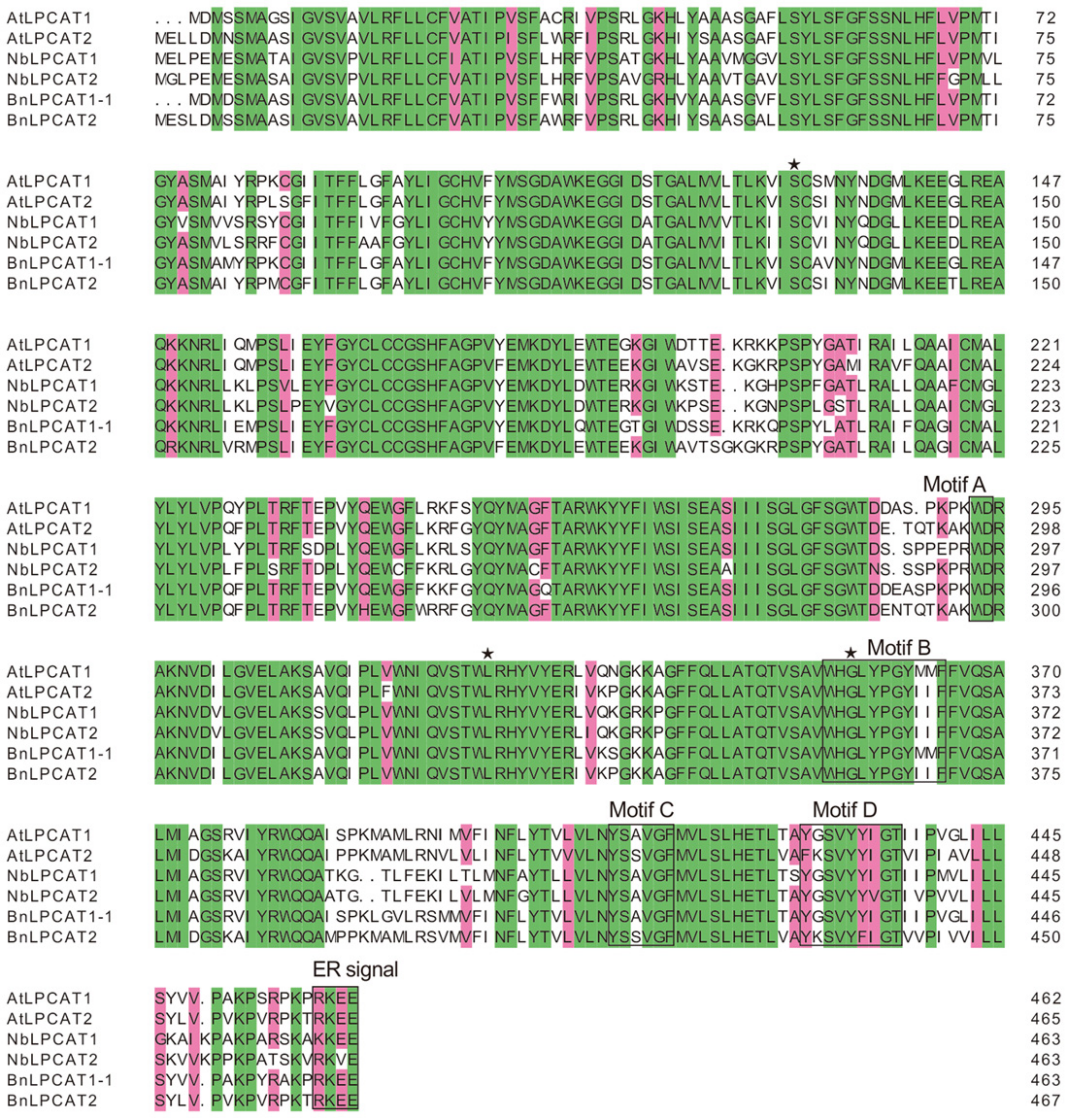
Sequence alignment of NbLPCATs with the related LPCATs from higher plants. The amino acid sequences of NbLPCATs were aligned, using the software Clustal X v1.83 with those of characterized LPCATs from *B*. *napus* and *A*. *thaliana*. The Jalview v2.8.2 program was used to highlight the homology between LPCAT protein sequences. Conserved motifs and the putative ER signal are boxed. Invariant residues are marked with black triangle stars.

**Fig 2:**
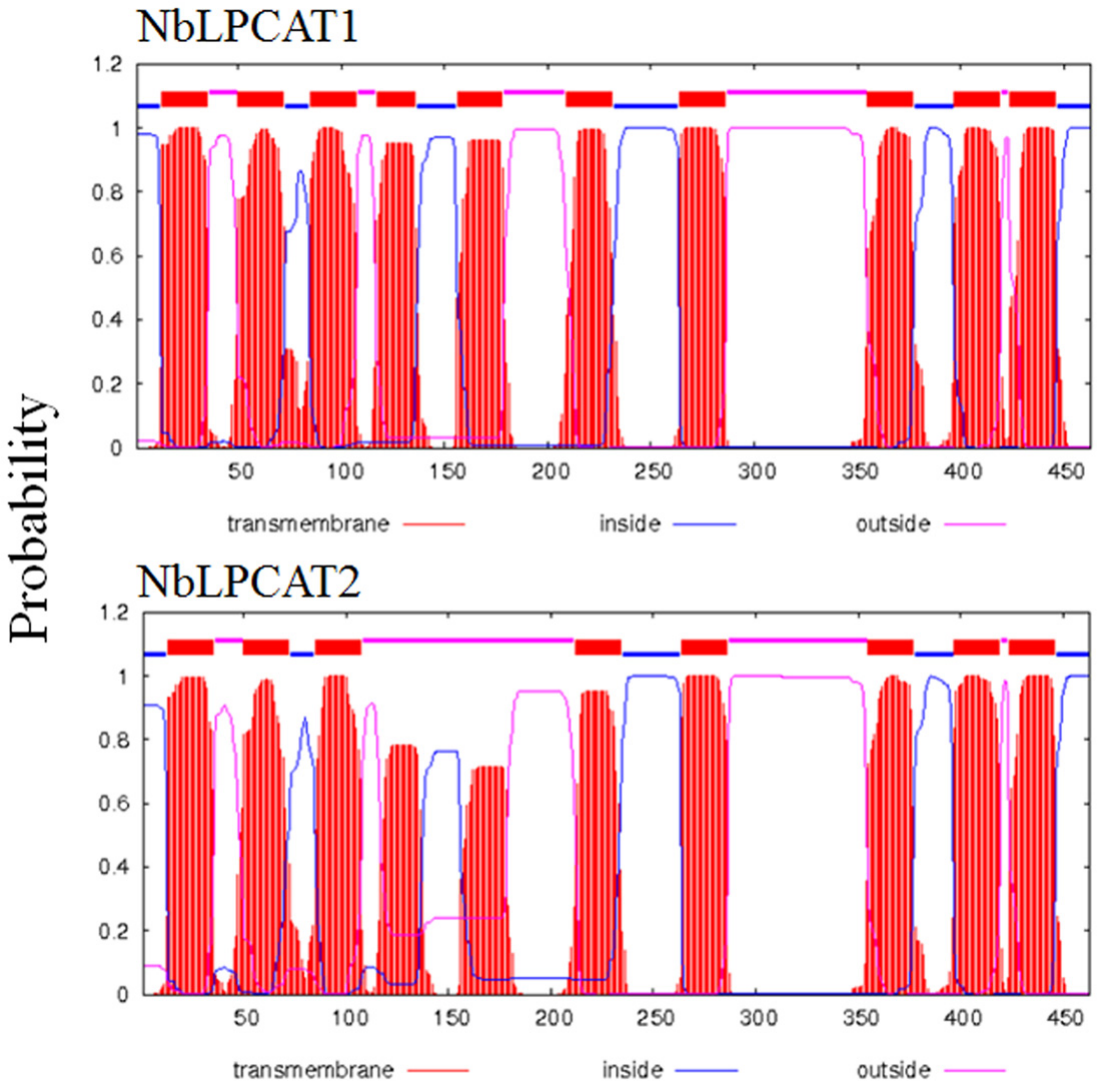
Predicted transmembrane domains for *N*. *benthamiana* LPCAT sequences. The TMHMM web tools of the Center for Biological Sequence Analysis, Technical University of Denmark TMHMM Server plot the probability of the ALDH sequence forming a membrane-spanning helix (0–1.0 on the y-axis). The transmembrane regions are shown in red, whereas regions of those sequences predicted to be located inside or outside the membrane are shown in blue and pink, respectively.

Putative cDNAs encoding *N*. *benthamiana* LPCATs, from 14-day-old seedlings, designated *NbLPCAT1* and *NbLPCAT2* were obtained by RT-PCR. Although a number of cDNAs encoding plant LPCATs have been cloned and characterized recently, *NbLPCAT1* and *NbLPCAT2* represent the first cDNAs encoding LPCAT enzymes from tobacco. The ORFs of both NbLPCAT1 and NbLPCAT2cDNAs predict a 463-aa protein that has a calculated molecular mass of 52.3 kDa and 52.0 kDa (predicted by Protparam, http://www.expasy.ch), respectively. These two NbLPCAT proteins have a predicted isoelectric point of 9.18 and 9.34, respectively, and thus are positively charged at neutral pH.The predicted proteinsNbLPCAT1 and NbLPCAT2exhibit limited (27%) sequence identity to the yeast LPCAT but high sequence identity to most reported LPCATs from plants, including *Brassica napus* (74%, Genbank Accession:CDY63224.1), *Oryza sativa* (74%, Genbank Accession:NP 001047723.1), *Arabidopsis thaliana* (78%, Genbank Accession:AAM13086.1), *Glycine max* (79%, Genbank Accession: XP 003525728.1), *Solanum tuberosum* (87%, Genbank Accession: XP 006358867.1), and *Ricinus communis* (79%, Genbank Accession: AGO14581.1). Alignment of the deduced amino acid sequences of NbLPCAT1 and NbLPCAT2 with other plant LPCATs revealed four highly conserved motifs (Fig 1), among which the former three motifs (Motifs A, B and C) are the previously defined classic functional motifs essential for lysophospholipid acyltransferase activities in the MBOAT family [32]. Although the motif D was not identified as the classic MBOAT motif (YxxxYFxxH), it is conserved among plant LPCATs, which might suggest a role in the recognition of lysoPC. Sequence analysis also predicted a conserved ER signal sequence (R(K)KE(V)E) at the end of C-terminus. At least three residues, Ser^132^, Leu^327^ and Gly^359^, which may be catalytic sites critical for lysophospholipid acyltransferase activities in the MBOAT family, remain invariant among all selected plant LPCATs. The amino acid sequences of NbLPCATs were also analyzed using TMHMM Server v.2.0 (http://www.cbs.dtu.dk/services/TMHMM/) which predicts transmembrane helices in proteins. NbLPCAT1 and NbLPCAT2 contain 10 and 8 strongly hydrophobic transmembrane regions (Fig 2), respectively, suggesting that both of them are typical transmembrane proteins. Together, NbLPCATs were predicted to be the endoplasmic reticulum-located membrane-spanning proteins sharing high sequence similarity with other plant LPCATs.

**Fig 3:**
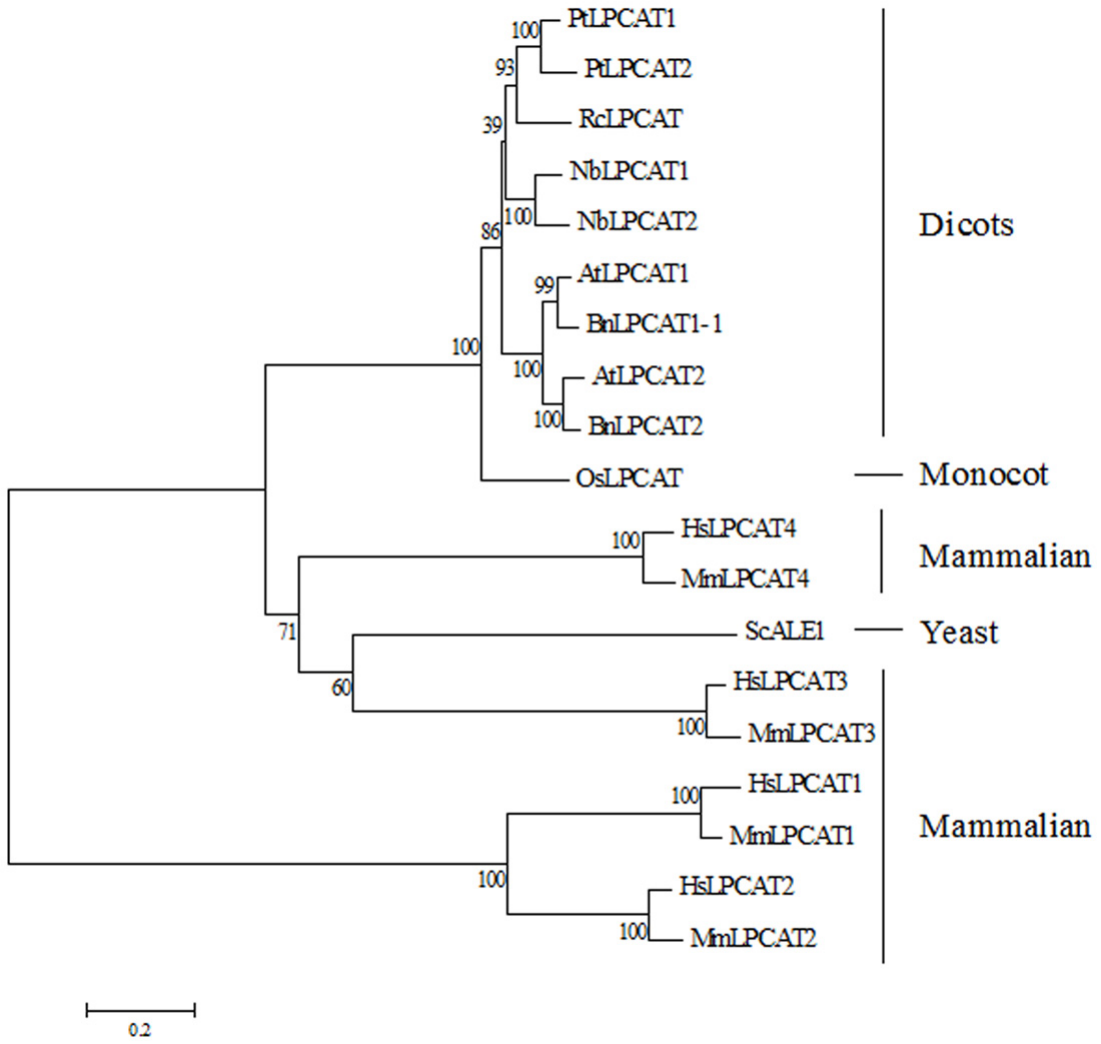
Phylogenetic relationship among deduced amino acid sequences of NbLPCAT1, NbLPCAT2 and LPCATs from other organisms. The tree was constructed according to the Neighbor-Joining algorithm. The percentages of bootstrap support, derived from 1000 replicates, are shown at branch points. AtLPCAT1 (*Arabidopsis thaliana*, GenBank accession number F4IDU4), AtLPCAT2 (*Arabidopsis thaliana*, GenBank accession number Q9CAN8), HsLPCAT1 (*Homo sapiens*, GenBank accession number BAE94688), HsLPCAT2 (*Homo sapiens*, GenBank accession number BAF47696), HsLPCAT3 (*Homo sapiens*, GenBank accession number NP_005759), HsLPCAT4 (*Homo sapiens*, GenBank accession number NP_620154), MmLPCAT1 (*Mus musculus*, GenBank accession number BAE94687), MmLPCAT2 (*Mus musculus*, GenBank accession number BAF47695), MmLPCAT3 (*Mus musculus*, GenBank accession number BAG12120), MmLPCAT4 (*Mus musculus*, GenBank accession number BAG12122), OsLPCAT (*Oryza sativa*, GenBank accession number Q6EP89), PtLPCAT1 (*Populus trichocarpa*, GenBank accession number B9GW66), PtLPCAT2 (*Populus trichocarpa*, GenBank accession number B9GKN7), RcLPCAT (*Ricinus communis*, GenBank accession number B9RC25), ScALE1 (*Saccharomyces cerevisiae*, GenBank accession number NP_014818).

Previous phylogenetic analysis of the acyltransferase family showed that LPCAT proteins from yeast, plant and animal grouped together and could be distinguished from wax synthase, diacylglycerol acyltransferase, and glycerol-3-phosphate acyltransferase [18]. To gain a picture of the evolution of LPCATs, we generated a phylogenetic tree that comprised 19 LPCAT polypeptides from yeast, mammals and seed plants including monocot and dicots. It appeared that LPCAT proteins from mammals (human and mouse) are clearly divided into two separate subclasses: one consists of LPCAT1 and LPCAT2, and the other contains LPCAT3 and LPCAT4. Moreover, mammalian LPCAT3 and yeast LPCAT grouped together with strong bootstrap support, suggesting a close phylogenetic relationship between them. The phylogenetic analysis also revealed that the polypeptide sequences of *N*. *benthamiana* LPCATs are phylogenetically more distant from LPCATs of *A*. *thaliana* and *B*. *napus* (Brassicaceae species) than those of *P*. *trichocarpa* (Salicaceae species) and *R*. *communis* (Euphorbiaceae species) (Fig 3). Although a strong conclusion cannot be made about the origin of plant LPCATs at present due to the lacking of available LPCAT sequence information, it could be seen that almost all LPCATs from dicots form a large clade, which may suggest a common phylogenetic origin of LPCATs from dicots.

### NbLPCATs preferentially incorporate PUFAs into PC *in vivo*

**Fig 4:**
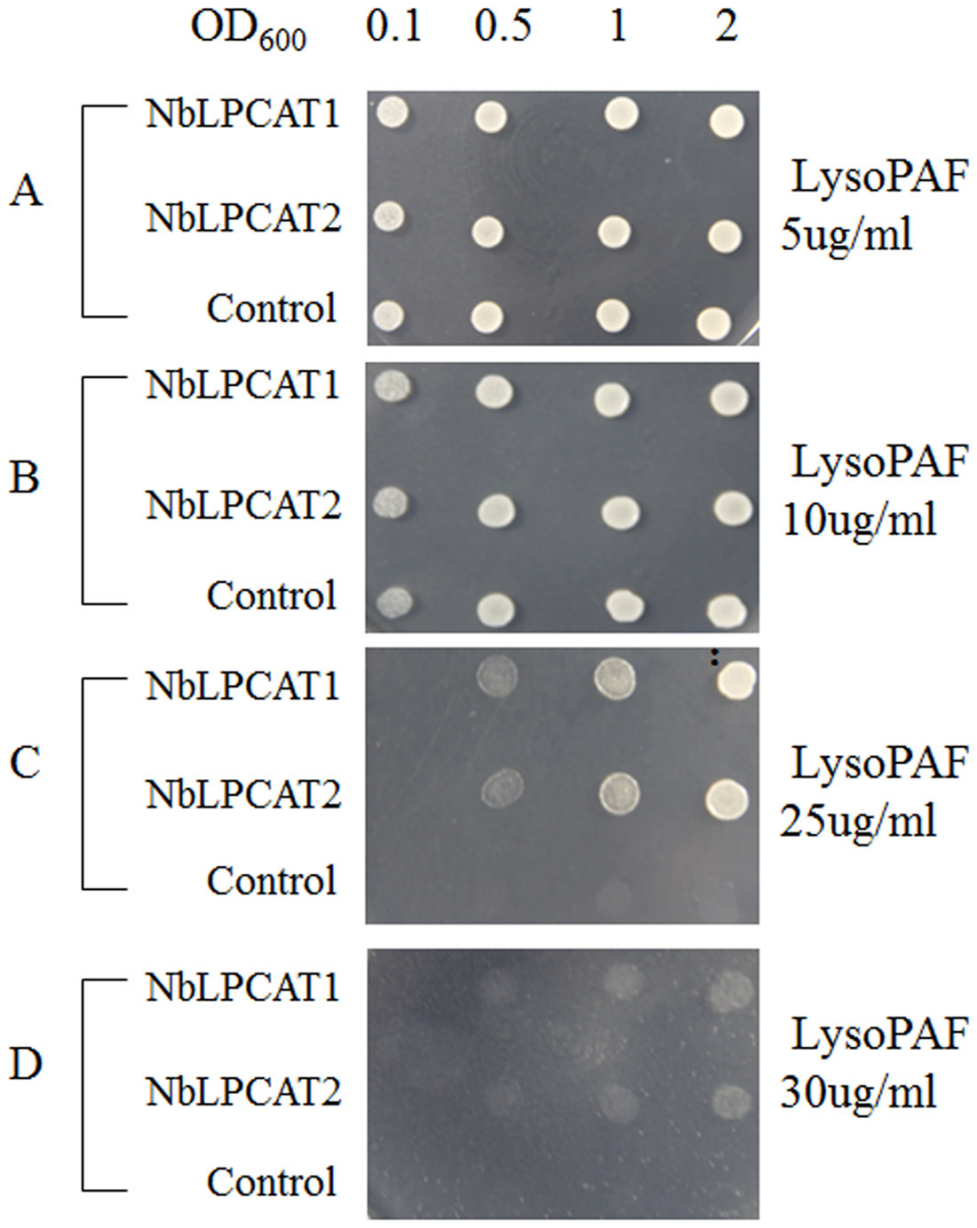
LysoPAF sensitivity test for yeast mutant Y02431 (*lca1*△) expressing NbLPCAT or harbouring the pESC-Ura empty vector. Yeast cells grown overnight and induced for the expression of NbLPCAT for 24 h were suspended in sterile distilled water and adjusted to an OD_600_ of 2, 1, 0.5, and 0.1. The resulting 2 μL yeast solution was spotted on a SC-Ura agar plate containing 5, 10, 25, and 30 μg/mL lysoPAF. The growth of yeast cells was evaluated after 72 h at 28°C.

To confirm the lysophopholipid:acyl-CoA acyltransferase activity of NbLPCAT1 and NbLPCAT2, heterologous expression was conducted in yeast. The constructs pESC-Ura/NbLPCAT1 and pESC-Ura/NbLPCAT2 containing full-length coding sequences of NbLPCATs and the empty vector (pESC-Ura) were transformed into the *S*. *cerevisiae* mutant strain Y02431 (*ale1* or *lca1*△). It was known that yeast has the ability to incorporate exogenously supplemented lysoPAF and reacylate this ether lipid within the cells [33]. However, the yeast *lca1*△mutant strain disrupted in endogenous LPCAT enzyme activity was hypersensitive to lysoPAF because reacylation of lysoPAF that was required for cell viability was blocked. To examine whether heterologously expressed NbLPCAT1 or NbLPCAT2 can alleviate or eliminate the toxicity of lysoPAF on the *lca1*△mutant yeast cells, we tested the growth of yeast transformants expressing NbLPCAT or containing empty vector. They were spotted onto agar plates containing different concentrations of lysoPAF. On plates containing 5 and 10 μg/mL lysoPAF, the *lca1*△mutant containing empty vector and expressing NbLPCAT1 and NbLPCAT2 can grow well because low concentration of lysoPAF could not inhibit the growth of yeast mutant cells (Fig 4A and 4B). Examination of the growth of the empty vector-introduced *lca1*△mutant strain on plates containing higher concentration of lysoPAF revealed a dose-dependent growth defect: growth was strongly impaired by 25 μg/mL lysoPAF whereas growth was completely blocked by 30 μg/mL lysoPAF. By comparison, the growth of the *lca1*△mutant transformed by NbLPCAT1 or NbLPCAT2 was restored to a great extent (Fig 4C and 4D). The restored growth of the *lca1*△mutant transformed by NbLPCAT1 or NbLPCAT2 was probably due to the reacylation of lysoPAF within the yeast cells upon the expression of a functional LPCAT enzyme, which could alleviate the toxicity of lysoPAF on yeast mutant cells. LysoPAF sensitivity test was a feasible method to confirm the phospholipid acyltransferase activity, by which several LPCAT genes have been identifiedin human [34], plants including *B*. *napus* [17] and *R*. *communis* [18].

**Fig 5:**
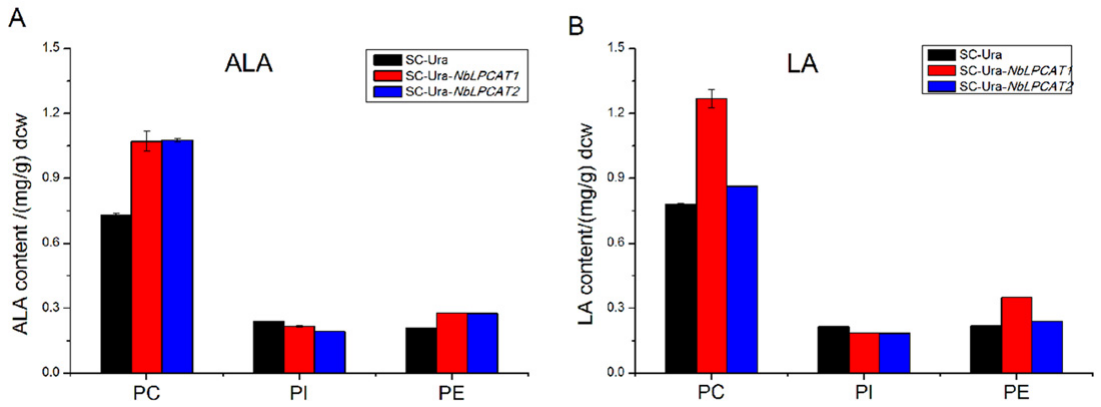
Distribution of exogenously supplemented ALA (A) or LA (B) in phospholipids from cultures of yeast mutant Y02431 (*lca1*△) expressing NbLPCAT or harbouring the pESC-Ura empty vector. Cultures were supplemented with 500 μM PUFA in the presence of 1% (w/v) Tween 40. The value was expressed as the amount of PUFA (mg, isolated from PC, PE, or PI) per g dry cell weight (mg/g). The data represent the mean±S.E. of three measurements. ALA, α-linolenic acid (18:3n3); LA, linoleic acid (18:2n6); PUFA, polyunsaturated fatty acid.

Since NbLPCATs have been shown to have LPCAT activity, we first used these yeast strains to determine substrate preference for PUFAs with *in vivo* functional assay. The *lca1*△mutant strains transformed with NbLPCAT1, NbLPCAT2 or empty vector were cultivated in the presence of exogenously added PUFA (LA or ALA), which was taken up by yeast and subsequently esterified into PUFA-CoA by the action of yeast acyl-CoA synthetases. Expression of *N*. *benthamiana* LPCAT in the *lca1*△ mutant cells allowed this enzyme to act upon PUFA-CoA and incorporated it into different lipid fractions. Analysis of phospholipid profiles of these yeast strains may provide insight into substrate preference of NbLPCATs. The expression of NbLPCAT1 resulted in more incorporation of ALA and LA into PC and PE when compared with that of the *lca1*△ mutant harboring empty vector, whereas no increase was observed in the amount of n3-PUFAs incorporated into PI (Fig 5A). In contrast, the expression of NbLPCAT2 led to higher incorporation of ALA than LA into PC, which was similar to those incorporated into PE. The amount of PUFAs incorporated into PI was also not increased in the recombinant yeast mutant expressing NbLPCAT2 when compared with the mutant harboring empty vector (Fig 5B). These data from *in vivo* assay indicate that *N*. *benthamiana* LPCATs can accept 18:2- and 18:3-CoAs as substrate and incorporate them into PC and PE.

### *In vitro* substrate specificity assays for NbLPCATs

**Fig 6:**
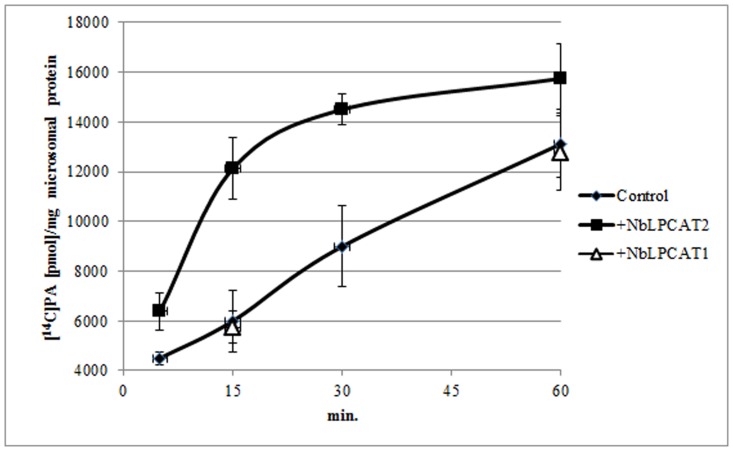
LPAAT activity (sn-1-18:1-LPA and^14^C-labeled 18:1-CoA as substrates) of microsomal preparation from yeast overexpressed with empty plasmid (control), NbLPCAT2 and NbLPCAT1.

**Fig 7:**
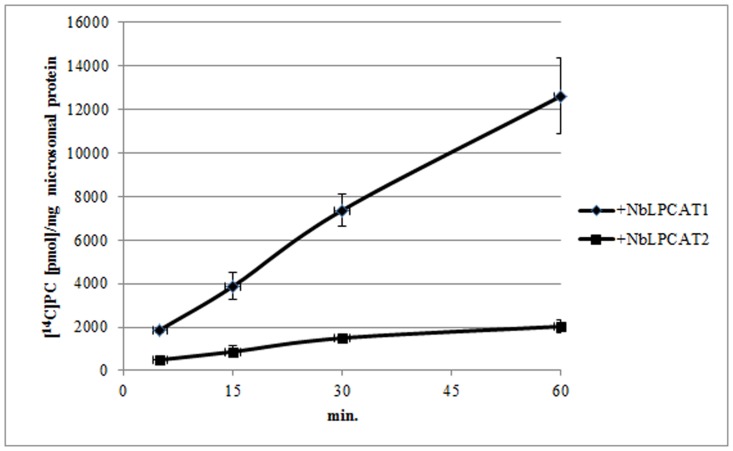
LPCAT activity (sn-1-18:1-LPC and^14^C-labeled 18:1-CoA as substrates) of microsomal preparation from yeast overexpressed with NbLPCAT1 and NbLPCAT2.

**Fig 8:**
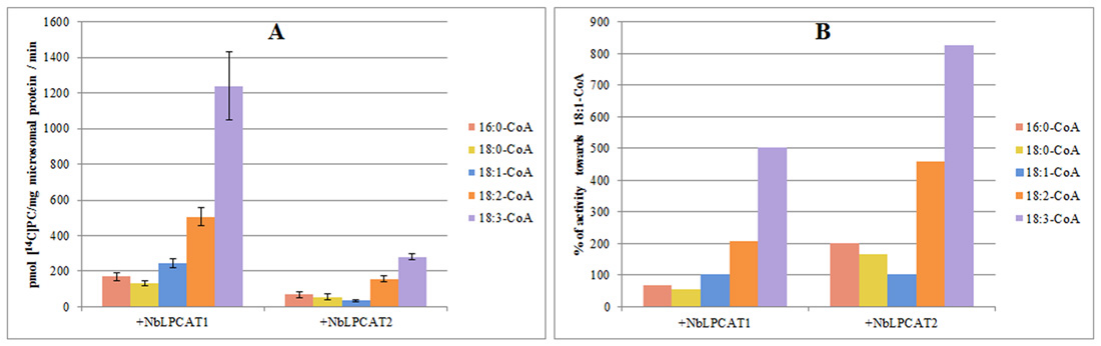
LPCAT activity of microsomal preparation from yeast overexpressed with NbLPCAT1 and NbLPCAT2 towards different acyl-CoAs. A, expressed as pmol of *de novo* synthesised [^14^C] PC/mg microsomal protein/min. B, expressed as % of activity towards 18:1-CoA.

**Fig 9:**
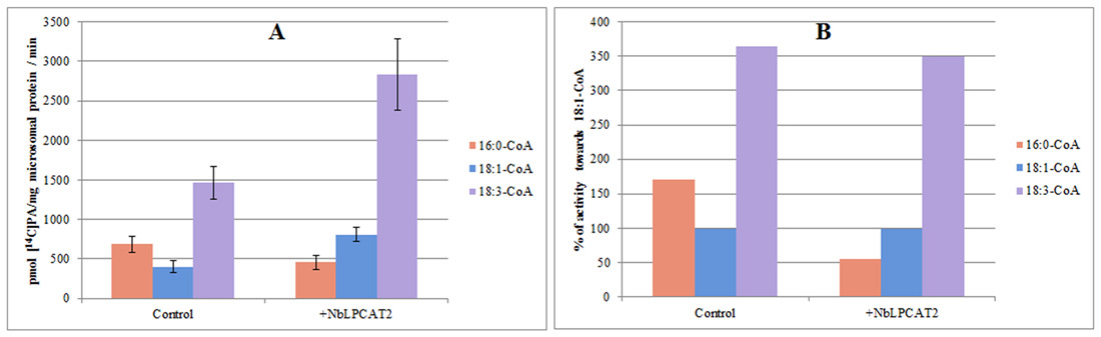
LPAAT activity of microsomal preparation from yeast overexpressed with empty plasmid (control) and with NbLPCAT2 towards different acyl-CoAs. A, expressed as pmol of *de novo* synthesised [^14^C] PA/mg microsomal protein/min. B, expressed as % of activity towards 18:1-CoA.

The substrate specificities of NbLPCATs were further examined with *in vitro* enzymatic activity assays. Lysophospholipid acyltransferase activity was determined by measuring the incorporation of radiolabeled acyl-CoAs into phospholipids. Using microsomal fraction (microsome) from the yeast *lca1*△mutant transformed with empty plasmid or each of NbLPCATs as enzyme source and ^14^C-labeled oleoyl-CoA as acyl donor, we first assessed the LPLAT activity of both NbLPCATs towards four different phospholipids including lysoPC, lysoPA, lysoPE, and lysoPS. Of the four lysophospholipids, the microsomal fractions from yeast mutant cells transformed with each NbLPCAT and empty plasmid all showed LPLAT activity towards lysoPA. The microsomal fraction extracted from yeast transformed with NbLPCAT1 exhibited almost equivalent LPAAT activity compared to that of control (transformed with empty plasmid), which appeared to be endogenous acyltransferase activity, whereas the microsomal fraction from yeast transformed with NbLPCAT2 demonstrated higher LPAAT activity than that from control and NbLPCAT1 (Fig 6). Since the yeast *lca1*△mutant transformed with empty plasmid was almost deficient in LPCAT activity, we then tested the LPCAT activity of both NbLPCATs using 18:1-lysoPC and ^14^C-labeled oleoyl-CoA as substrates. The LPCAT activity for the microsomal fraction from yeast transformed with NbLPCAT1 increased linearly with reaction time, while it was only detectable for microsomal fraction from yeast transformed with NbLPCAT2 (Fig 7). Using *sn*-1-18:1-lysoPC as acyl acceptor, we further analyzed acyl-CoA selectivity of both NbLPCATs. Among the fatty acyl-CoAs tested, 18:3-CoA had the highest LPCAT activity for both NbLPCATs compared with other acyl-CoAs, indicating a clear preference of both NbLPCATs for 18:3-CoA. In this assay, NbLPCAT1 also exhibited the LPCAT activity towards other acyl-CoAs with the order of 18:2>18:1>16:0>18:0, while the acyl-CoA preference of NbLPCAT2 was 18:2>16:0>18:0>18:1 (Fig 8A and 8B). Since NbLPCAT1 showed almost undetectable LPAAT activity, we finally evaluated acyl-CoA selectivity of NbLPCAT2 with the LPAAT activity using *sn*-1-18:1-lysoPA as the substrate. Both microsomal fractions extracted from yeast cells transformed with NbLPCAT2 and empty plasmid demonstrated the highest LPAAT activity towards 18:3-CoA compared with 18:1-CoA and 16:0-CoA (Fig 9A and 9B). Of the acyl-CoAs tested, 18:3-CoA was also the preferred acyl-CoA for the LPAAT activity of NbLPCAT2. Based on the results of substrate specificity, we concluded that NbLPCAT2 is probably a LPAAT enzyme although it also showed the relatively low LPCAT activity.

In the previous studies, *in vitro* enzymatic assays were performed to analyze substrate specificities of LPCATs using diverse lysophopholipids as acceptors and diverse acyl-CoAs as donors. For example, using yeast microsome as enzyme source of LPCAT, Zheng et al. [17] found that *B*. *napus* LPCATs (BnLPCAT1-1 and BnLPCAT2) showed higher acyltransferase activities with lysoPC compared to lysoPA and lysoPE, and had a preference for 16:0-lysoPC, 18:0-lysoPC and 18:1-lysoPC. Using lysoPC as acceptor, they also found that *B*. *napus* LPCATs exhibited a preference for unsaturated fatty acyl-CoAs including 16:1-, 18:1-, 18:2-, and 18:3-CoAs. It has been known that plant LPCATs had very similar acyl specificities for C_18_-unsaturated acyl-CoAs in the forward reaction [14]. Our *in vivo* functional assay in yeast cells demonstrated that NbLPCATs could incorporate exogenously supplemented 18:2 and 18:3 into PC, which provides indirect evidence on substrate preference for unsaturated C_18_-CoAs and lysoPC. The results from *in vivo* functional assay were consistent with the results from *in vitro* enzymatic assay of both NbLPCATs. However, acyl-CoA preference of *N*. *benthamiana* LPCATs appears to be different from that of other plant LPCATs. For example, *B*. *napus* LPCATs exhibited similar LPCAT activity when 18:3-CoA, 18:2-CoA, 16:1-CoA, and 18:1-CoA were used acyl donor [17]; whereas *R*. *communis* LPCAT (RcLPCAT) used monounsaturated acyl-CoAs (16:1-CoA and 18:1-CoA) as the preferred substrates [18]. In contrast, our *in vitro* assay demonstrated that both *N*. *benthamiana* LPCATs showed a clear preference for 18:3-CoA. Nevertheless, our *in vivo* functional assay and *in vitro* enzyme assay both provide a basis for determining the role of LPCATs in mediating the flux of polyunsaturated fatty acid intermediates when yeast or tobacco is considered to be used as transgenic hosts.

### Tissue-specific expression of NbLPCATs

**Fig 10:**
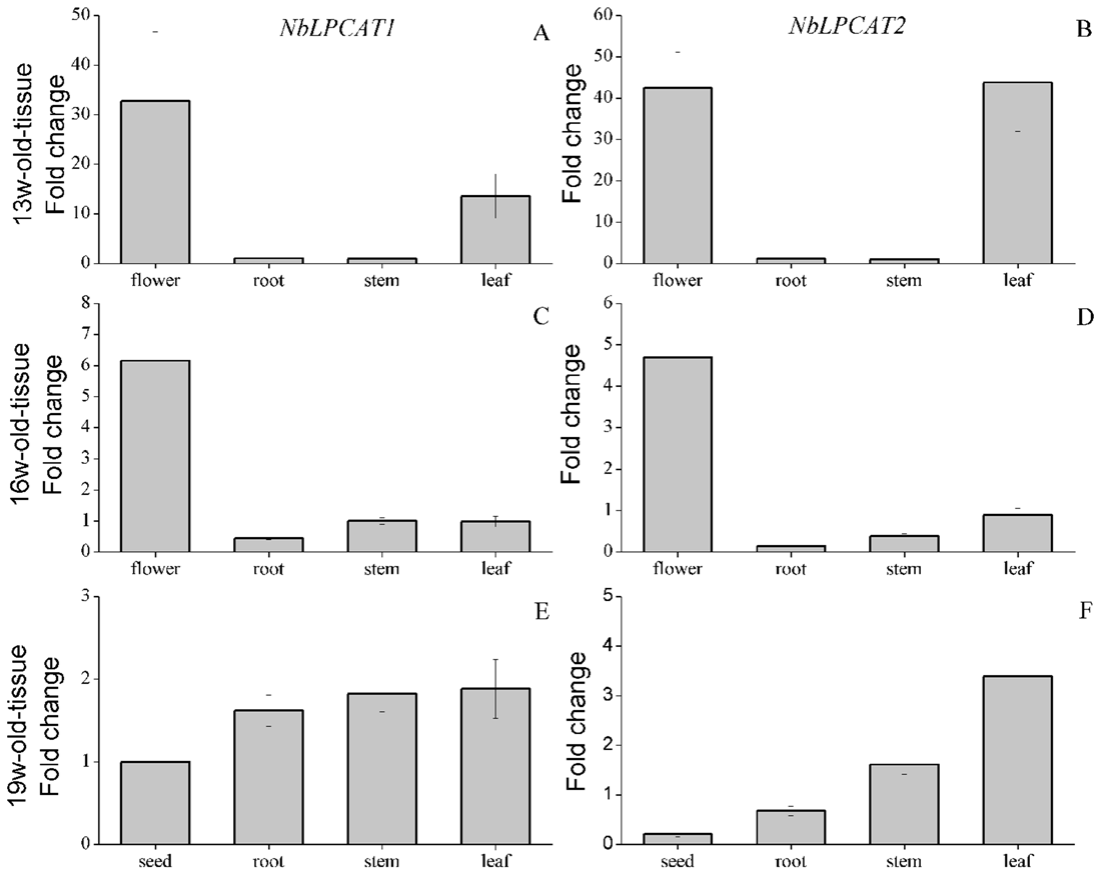
Tissue-specific expression patterns of *NbLPCAT* genes in different tissues of 13-week-old (A, B), 16-week-old (C, D), and 19-week-old (E, F), tissue-cultivated *N*. *benthamiana*. The expression levels of *NbLPCAT*s were analyzed by the real-time quantitative RT-PCR method. X-axis indicates different tissues and y-axis indicates relative expression levels. Gene encoding elongation factor 1α signal was used as the reference gene. Error bars represent standard deviations of mean value from three technical replicates. W, week.

**Fig 11:**
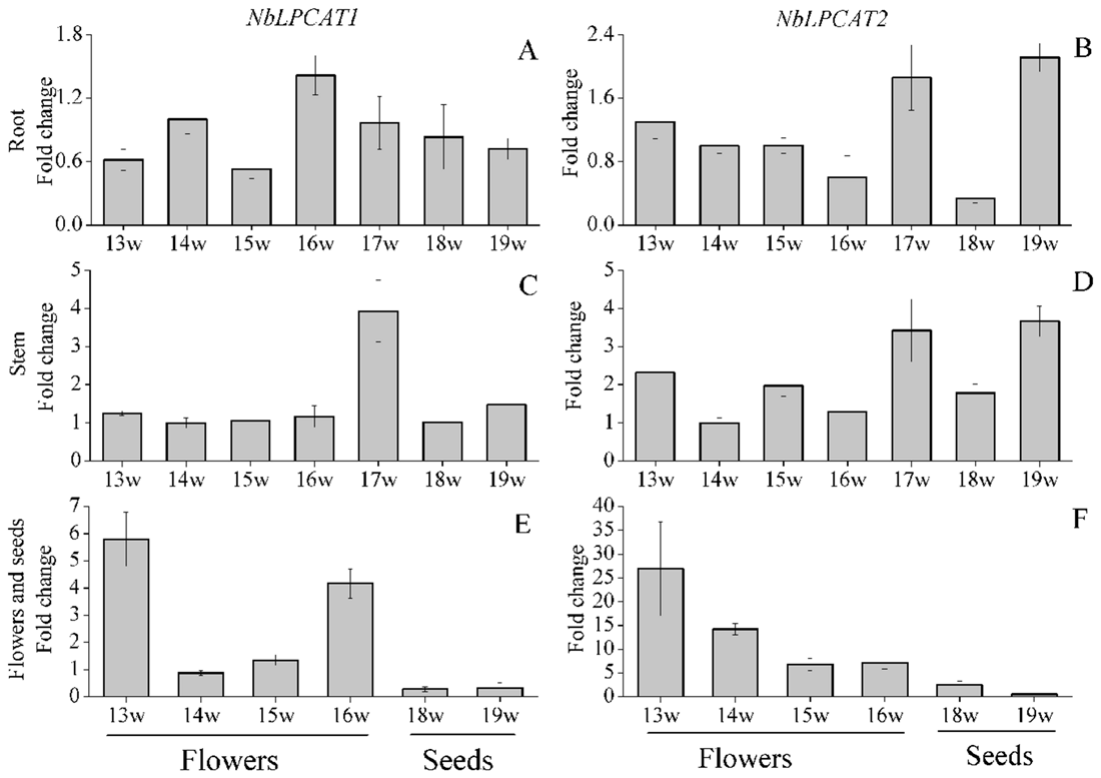
Expression patterns of *NbLPCAT* genes in roots (A,B), stems (C,D), flowers and seeds (E,F) during different developing periods of tissue-cultivated *N*. *benthamiana*. The expression levels of *NbLPCAT*s were analyzed by the real-time quantitative RT-PCR method. X-axis indicates different developing periods and y-axis indicates relative expression levels. Gene encoding elongation factor 1α signal was used as the reference gene. Error bars represent standard deviations of mean value from three technical replicates. W, week.

It has been shown that LPCAT may participate in lipid metabolism in plants [17, 18]. In order to understand the roles of NbLPCATs in lipid metabolism and development, we examined the tissue-specific expression profiles of each of NbLPCATs by real-time fluorescent quantitative RT-PCR. Each gene was analyzed using RNA from roots, stems, flowers and developing seeds at different developing phases, and 13 w-, 16 w-old roots, stems, leaves and flowers, as described in “Materials and methods.” Both *NbLPCAT1* and *NbLPCAT2* shared similar expression patterns at 13 w and 16 w in different tissues. *NbLPCAT1* and *NbLPCAT2* were predominantly expressed in flowers and leaves at 13 w (Fig 10A and 10B), while they were mainly expressed in flowers at 16 w (Fig 10C and 10D). *NbLPCAT1*was extensively expressed in seeds, roots, stems and leaves at 19 w, while *NbLPCAT2* showed predominant expression in stems and leaves at this growth phase (Fig 10E and 10F). Examination of expression levels of NbLPCATs at different time-points in a specific tissue showed that both *NbLPCAT1* and *NbLPCAT2* were expressed from 13 w to 19 w in roots, and in stems their transcripts accumulated throughout stem development with a peak at 17 w or 19 w (Fig 11A, 11B, 11C and 11D).The expression level of *NbLPCAT1* was similar to that of *NbLPCAT2* in seeds, and weak expression was found at both 18 w and 19 w. In flowers their transcript levels peaked at 13 w, and the transcript of *NbLPCAT1* was declined at 14 w and 15 w, and then up-regulated at 16 w; whereas the transcript level of *NbLPCAT2* was gradually declined after 13 w (Fig 11E and 11F). These findings indicate a strong expression of *NbLPCAT*s at early flower maturing phase, which might suggest a functional importance of *NbLPCAT*s in tobacco flower development and maturation. In the analysis of tissue-specific expression profiles of NbLPCAT genes, we examined whether the developmental regulation of *NbLPCAT* expression had contribution to seed oil synthesis and accumulation in tobacco. It was found that strong expression of *NbLPCAT*s in flowers preceded oil accumulation in seeds. There is unlikely to establish a relationship between oil accumulation and *LPCAT* function in tobacco seeds, because *NbLPCAT*s were expressed at low levels in seeds compared with other tissues at the same time-point(Fig 10E and 10F) and throughout seed development (Fig 11E and 11F). Tissue expression analyses of *LPCAT*s have also been performed in other plants. Different from the expression pattern of *N*. *benthamiana LPCAT*s, *B*. *napus LPCAT BnLPCAT1-1* exhibited higher expression level than *BnLPCAT2* in the examined tissues, and the highest transcript level of *BnLPCAT1-1* was observed in young seedlings, followed by leaves, buds and flowers [17]. In the castor plant *R*. *communis*, although strong expression of *RcLPCAT* was found in developing seeds when compared with other vegetative organs and reproductive tissues, the expression of *RcLPCAT* was not regulated throughout seed development [18]. Because we observed the ubiquitous expression of *NbLPCAT*s in diverse tissues of *N*. *benthamiana*, a general role of *NbLPCAT*s was suggested for lipid synthesis and plant development in tobacco.

## Conclusions

Two putative LPCAT proteins identified from *N*. *benthamiana* share high sequence similarity with other plant LPCATs and have several typical signature motifs and invariant residues of the membrane bound *O*-acyltransferase family. Heterologous expression of *N*. *benthamiana* LPCATs in the yeast *lca1*△mutant not only confirmed their lysophospholipid acyltransferase activity but also suggested their substrate selectivity. Similar to LPCATs from other plants such as *R*. *communis* and *B*. *napus*, NbLPCAT1 shows a preference for lysoPC against other lysophospholipids, and preferentially uses C18-polyunsaturated fatty acyl-CoA as substrates. However, NbLPCAT2 exhibits higher LPLAT activity towards lysoPA than lysoPC, indicating that NbLPCAT2 is probably a LPAAT enzyme although it also has a weak LPCAT activity. Based on tissue-specific gene expression patterns, we found that the highest expression level of *N*. *benthamiana* LPCAT genes was detected in flowers, while *R*. *communis* LPCAT was highly expressed in roots, stems and developing seeds, and the highest expression level of *B*. *napusLPCAT1-1* was observed in early seedlings. The difference of expression levels of plant LPCAT genes in various tissues may indicate different roles of LPCAT genes in plant development and lipid metabolism. The molecular characterization of *N*. *benthamiana* LPCATs paves the way for a better understanding of PC remodeling that is crucial for the synthesis of biological membranes in tobacco.
